# Schizophrenia polygenic risk is associated with child mental health problems through early childhood adversity: evidence for a gene–environment correlation

**DOI:** 10.1007/s00787-021-01727-4

**Published:** 2021-02-26

**Authors:** Koen Bolhuis, Lisa R. Steenkamp, Laura M. E. Blanken, Alexander Neumann, Philip R. Jansen, Manon H. J. Hillegers, Charlotte A. M. Cecil, Henning Tiemeier, Steven A. Kushner

**Affiliations:** 1 Department of Child and Adolescent Psychiatry, Erasmus Medical Center, Sophia Children’s Hospital, University Medical Center Rotterdam, Rotterdam, The Netherlands; 2 Generation R Study Group, Erasmus Medical Center, Rotterdam, The Netherlands; 3 Lady Davis Institute for Medical Research, Jewish General Hospital, Montreal, QC Canada; 4 Department of Complex Trait Genetics, Center for Neurogenomics and Cognitive Research, Amsterdam Neuroscience, VU University, Amsterdam, The Netherlands; 5 Department of Clinical Genetics, Amsterdam University Medical Centre, Location VUmc, Amsterdam, The Netherlands; 6 Department of Epidemiology, Erasmus Medical Centre, Rotterdam, The Netherlands; 7 Molecular Epidemiology, Department of Biomedical Data Sciences, Leiden University Medical Center, 2333 ZC Leiden, The Netherlands; 8 Department of Social and Behavioral Sciences, Harvard T.H. Chan School of Public Health, Kresge Building, Room 619, 677 Huntington Avenue, Boston, MA USA; 9 Department of Psychiatry, Erasmus University Medical Center, Rotterdam, The Netherlands

**Keywords:** Generation R, Psychosis, Gene–environment, Stressful life events, Population-based

## Abstract

Previous studies have shown that schizophrenia polygenic risk predicts a multitude of mental health problems in the general population. Yet it is unclear by which mechanisms these associations arise. Here, we explored a possible gene–environment correlation in the association of schizophrenia polygenic risk with mental health problems via childhood adversity. This study was embedded in the population-based Generation R Study, including *N* = 1901 participants with genotyping for schizophrenia polygenic risk, maternal reporting of childhood adversity, and Child Behaviour Checklist measurement of mental health problems. Independent replication was attempted in the Avon Longitudinal Study of Parents and Children (ALSPAC; *N* = 3641). Associations were analysed with Poisson regression and statistical mediation analysis. Higher burden of schizophrenia polygenic risk was associated with greater exposure to childhood adversity (*P*-value threshold < 0.5: Generation R Study, OR = 1.08, 95%CI 1.02–1.15, *P* = 0.01; ALSPAC, OR = 1.02, 95%CI 1.01–1.03, *P* < 0.01). Childhood adversities partly explained the relationship of schizophrenia polygenic risk with emotional, attention, and thought problems (proportion explained, range 5–23%). Direct effects of schizophrenia polygenic risk and adversity on mental health outcomes were also observed. In summary, genetic liability to schizophrenia increased the risk for mental health problems in the general paediatric population through childhood adversity. Although this finding could result from a mediated causal relationship between genotype and mental health, we argue that these observations most likely reflect a gene–environment correlation, i.e. adversities are a marker for the genetic risk that parents transmit to children. These and similar recent findings raise important conceptual questions about preventative interventions aimed at reducing childhood adversities.

**Supplementary Information:**

The online version contains supplementary material available at 10.1007/s00787-021-01727-4.

## Introduction

Multiple studies have reported robust associations of childhood adversity with psychotic symptoms and psychotic illness [1]. In particular, previous results have suggested a causal link between childhood adverse life events and psychotic symptoms [2]. However, others have argued that this relationship might be more complex and potentially explained by other composite risks, such as genetic liability [3].

Recent genome-wide association studies (GWAS) have contributed to an improved understanding of the genetic aetiology of psychotic disorders [4]. Polygenic risk scores are derived as the sum of single nucleotide polymorphism (SNP) dosages weighted by effect directions and sizes obtained from GWAS results. These scores are widely used as a metric for additive genetic liability of a given trait or disease, including schizophrenia. Although currently available polygenic risk scores have limited clinical utility for diagnosis of schizophrenia due to low predictive power [5], they have proven very useful in etiological research. Several studies have employed schizophrenia polygenic risk scoring to investigate developmental manifestations of schizophrenia genetic liability in the general population, including early-life emotional and behavioural problems and cognition [6–9].

We have previously reported that increased schizophrenia polygenic risk is associated with a higher burden of childhood emotional, attention, and thought problems [10]. However, the mechanisms through which polygenic scores for schizophrenia associate with these early-life phenotypes has remained poorly understood. Genotype likely exerts a direct effect on behavioural outcomes [11, 12], but this relationship may be at least partially explained by environmental exposures, such as childhood exposure to adversity. Children grow up in environments which are partially determined by their parents’ genotypes, leading to a correlation between a child’s genotype and their environment [13]. The notion that a child’s environment can in part be explained by their genotype is widely acknowledged. Twin and family studies have indicated substantial contributions of children’s genotype to factors which are typically understood as environmental, such as parenting, social support and life events [14, 15]. These associations are typically referred to as gene–environment correlations, of which different types can be distinguished [13, 16]. Passive gene–environment correlation refers to associations between a child’s genotype and the behaviour of genetically related individuals, as has been observed for parenting [17]. Evocative gene–environment correlation occurs when a child’s genetically driven behaviour elicits responses from others, such as punishment [18]. Active gene–environment correlation occurs when children’s life experiences are directly influenced by their own genetically determined behaviours, such as thrill-seeking.

Twin and SNP-heritability studies have demonstrated that childhood adversities are to some degree determined by a child’s genetics [14]. Non-zero heritability of environmental exposures would indicate that the “environmental” risk effect on child psychopathology is, at least in part, genetically determined [16]. Only a few studies have employed schizophrenia polygenic risk scores to study the possibility of such gene–environment correlations. Increased schizophrenia polygenic risk has been associated with higher paternal age [18], increased likelihood to be adopted [19], and more bullying victimisation [20], each of which is suggestive of a gene–environment correlation [16, 21]. Moreover, a recent study found an association between schizophrenia polygenic risk and exposure to trauma in childhood [22], but the extent to which exposure to adversity explains the relationship between schizophrenia liability and mental health problems remains to be further explored.

In the current study, we aimed to determine whether the previously reported associations between schizophrenia polygenic risk and mental health problems might be partly explained through exposure to childhood adversity [7, 10]. Furthermore, we calculated the SNP-heritability of childhood adversity to determine the extent to which it is influenced by common genetic variants [14, 15]. As the SNP-heritability captures the joint effect of all measured genetic variation, it provides context to the polygenic score results by providing a theoretical maximum the gene–environment effects could achieve. Lastly, we examined the specificity of the relationship between schizophrenia polygenic risk and early-life adversity by also examining the association of polygenic risk for depression with early-life adversity. We performed these analyses in a population-based birth cohort from the Netherlands with replication attempted in an independent birth cohort from the United Kingdom.

## Methods

### Study population

The primary analyses of the present study were embedded within the Generation R Study, a prospective population-based birth cohort, which included 9778 pregnant women living in Rotterdam, the Netherlands [23]. The aim of the Generation R Study is to identify early genetic and environmental risks that influence maternal and child health and development. For this study, 2512 children of Western European descent (based on genetic ancestry; 53% of *n* = 4780 participants of European descent who were eligible for the age ten assessment) had genotype data available which passed quality control procedures (Supplemental Figure S1). Of these children, 1901 had information available on childhood adversities and mental health problems, which were assessed at mean age ten years. Study protocols were approved by the Medical Ethics Committee of the Erasmus Medical Centre. All participants and their mothers provided assent and informed consent, respectively.

### Attrition analysis

Comparisons were made between the final study sample (*N* = 1901) and participants who were genotyped and of European ancestry but with missing phenotype and environment data at mean age ten years (*N* = 2512). These groups did not differ in the proportion of girls (50.3% vs 46.3%, *P* = 0.09). The sample with missing data had 0.06SD higher scores of schizophrenia polygenic risk (*P* = 0.16). Children with complete data were more likely to have mothers with higher educational levels (73.4% vs 68.6%, *P* = 0.03).

### Genotyping, quality control, and polygenic risk scoring

Genotype quality control procedures for the Generation R cohort have previously been described [24]. Genotype data were collected either from cord blood at birth (Illumina 610K Quad Chip) or venapuncture during a visit to the research center (Illumina 660k Quad Chip). Variants were included if they passed sample (≥ 97.5%) and SNP call rates (≥ 95%), minor allele frequency ≥ 1%, and without significant deviation from Hardy–Weinberg disequilibrium (*P* < 10^–7^). In addition, individuals were screened for excess heterozygosity, sex mismatch, relatedness, and missing data. Individuals of European descent were selected within four standard deviations on the initial four dimensions through multidimensional scaling (MDS) analysis of the HapMap Phase II Northwestern European (CEU) population. Principal components of ancestry used as covariates in this study were based on the European-descent sample. Genotypes that passed quality control were prephased with the SHAPEIT software package [25]. Phased haplotypes were imputed using IMPUTE v2 [26] against the 1000 Genomes (phase I version 3) as the reference panel.

### Polygenic risk scoring

Common genetic risk variants associated with schizophrenia were obtained from the Psychiatric Genetics Consortium meta-analysis of case–control genome-wide association study (GWAS) of 33,640 cases and 43,456 controls [4] and depression polygenic risk scores were obtained from the most recent GWAS of 135,458 cases and 344,901 controls [27]. SNPs were clumped according to linkage disequilibrium (LD) to obtain the most significant SNP per LD-block (kilobase pair window: 250, LD r2 < 0.1), in line with earlier work in Generation R [10]. Polygenic scores were computed using PRSice [28], which implements a weighted mean for risk-associated alleles by the SNP effect size. Polygenic risk scores were standardized to a mean of 0 and standard deviation of 1 to facilitate interpretation. *P*-value thresholds for inclusion of SNPs in polygenic risk scores varied between *P* < 0.0005 and *P* < 1.0 in the Generation R Study and between 0.01 and 1.0 in the ALSPAC Study. Our primary analysis was performed at *P* < 0.5, a default cut-off established previously [10].

### Child emotional and behavioural problems

Emotional and behavioural problems were assessed at age ten years using the Child Behavior Checklist/6‐18 (CBCL), an internationally validated and reliable measure of emotional and behavioural problems on a continuous severity scale [29]. The CBCL/6‐18 consists of internalising (i.e., emotional) and externalising (i.e., behavioural) problems broadband scales. The internalising problems scale comprised the anxious/depressed, withdrawn/depressed, and somatic problems sub-scales. The externalising problems scale comprised the rule‐breaking and aggressive behaviour sub-scales. Items were reported by mothers on a three‐point scale based on behaviour of the prior 6 months.

### Childhood adversities

At mean age ten years, children and their mothers were invited to the research centre, where mothers were interviewed about their offspring’s childhood adversities [30]. The maternal interview was based on earlier work [30, 31], including questions on stressful life events and long-term difficulties. In case of an affirmative response, the child’s age when the event occurred was registered, and the perceived severity of each event was rated as ‘none’, ‘a little’, ‘moderate’, or ‘a lot’. Only events of at least ‘moderate’ severity were coded as adversities in the present analyses. In a first step, all adversities were summed to deal with low numbers of individual adverse events, which is in line with earlier work [30]. In additional analyses, we distinguished person-related (e.g., high workload at school, maltreatment) from environment-related adversities in childhood (e.g., neighbourhood problems, family financial difficulties) [30], as well as adversities occurring before age five years and adversities occurring after age five years. Mothers reported on their child’s adversities and mental health problems when the child was approximately ten years old. Mothers who reported their child having a higher burden of emotional and behavioural problems at ten years could be more likely to also retrospectively report greater severity of earlier adversities experienced by the child [32]. Therefore, we reasoned that inclusion of adversities with a severity rating of ‘moderate’ or ‘a lot’ could potentially introduce reverse causality through rater-error bias, for which we performed sensitivity analyses with additional adjustment for emotional and behaviour problems assessed with the CBCL at child age three years. The lifetime prevalence of adversities, and their categorization as person-related or environment-related, are provided in Supplementary Table S1.

### Replication sample

Data from the Avon Longitudinal Study of Parents and Children (ALSPAC) was used for replication. The initial ALSPAC cohort included 14,062 children born to women residing in Avon, United Kingdom, with an expected delivery date between 1 April 1991 and 31 December 1992 [33]. In total, 3641children had complete data on genotypes and cumulative childhood life events until age nine years. See supplemental material for more detailed information on genotyping and measures.

### Statistical analyses

The association of schizophrenia polygenic risk score with childhood adversities was performed using Poisson regression models. Models were adjusted for child age, sex, and four genetic principal components of genetic ancestry. Our analysis was planned such that only if an association of schizophrenia polygenic risk with the total score of childhood adversities was evident would we proceed to implement in-depth analyses of polygenic risk for person-related and environment-related adversities, as well as adversities that occurred before versus after age 5 years. Similarly, replication would be sought in the ALSPAC cohort only if a significant association between polygenic risk score and childhood adversities was found within the Generation R cohort. We performed sensitivity analyses with additional adjustment for CBCL total problems scores at age three years. All analyses were conducted using R statistical software.

**Fig. 1:**
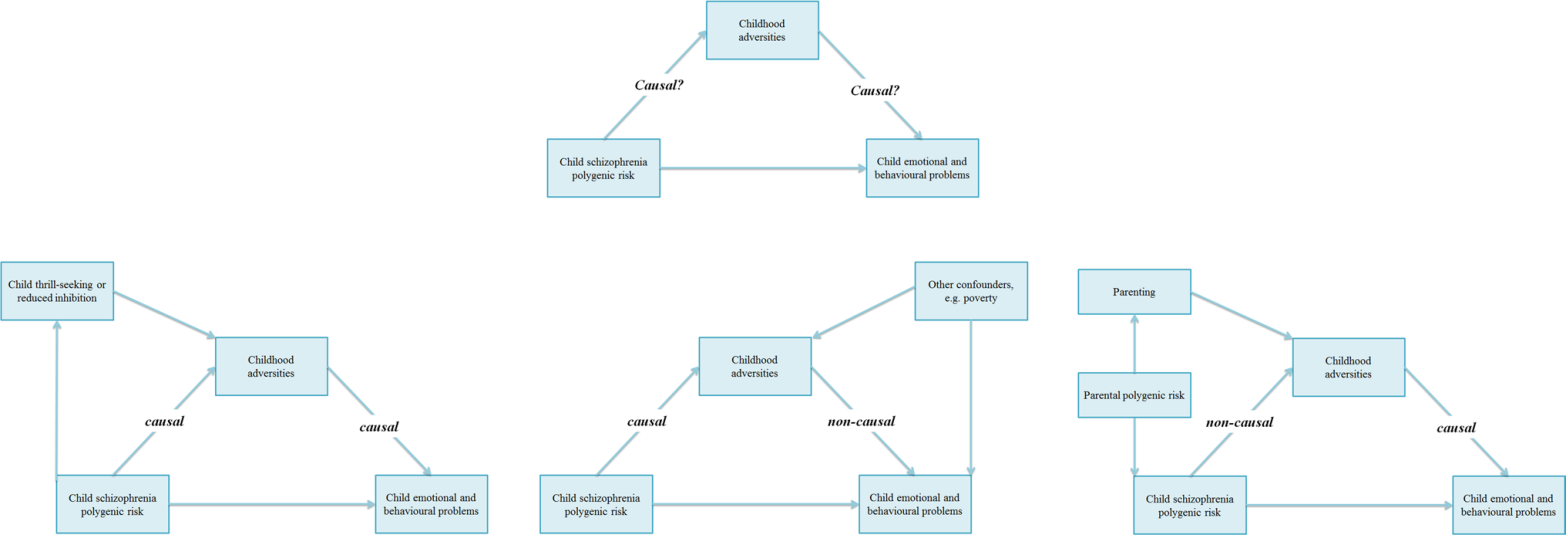
Conceptual model with potential explanatory mechanisms (lower panels **a**, **b**, and **c**). *Note*. Conceptual model of how childhood adversities might play a role in the association of schizophrenia polygenic risk with child emotional and behavioural problems (*upper panel*). The lower panel represents three potential explanatory mechanisms which might underlie this association. Left (**a**), a causal mediation framework, in which increased schizophrenia polygenic risk leads to more child emotional and behavioural problems through childhood adversities. Middle (**b**), a non-causal model in which the association between schizophrenia polygenic risk and child emotional and behavioural problems is not explained by childhood adversities, but by other confounding factors such as socioeconomic minority status. Right (**c**), a non-causal gene–environment correlational framework, in which the relationship between schizophrenia polygenic risk and child emotional and behavioural problems is explained by childhood adversities, but which in turned are determined by other factors such as parenting and parental genetic factors

Statistical mediation analyses were conducted to explore the extent to which the prospective association between schizophrenia polygenic risk and child mental health problems was explained by early-life adversity (Fig. 1). It was not our aim to infer causality as we cannot distinguish between passive, evocative or active gene–environment correlation and, thus, the statistical co-variation cannot be separated into mediation or confounding (lower panel of Fig. 1, figures left to right). First, separate linear regression analyses were performed for the associations between: (1) exposure (child polygenic risk score) and outcome (child emotional and behavioural problems), i.e. the total effect; (2) exposure and mediator (childhood adversities), i.e. the gene–environment association; (3) mediator and outcome controlling for exposure, i.e. the direct effect. If all three associations were significant, statistical mediation analyses were conducted in one single model to obtain the mediated/co-varied effect. In case of significant mediation/co-variation, the proportion by which the exposure-outcome estimate was attenuated after inclusion of the covariate was calculated. These analyses were performed using the *mediation* package in R [34]. Again, a hierarchical approach was employed; no subsequent test of a syndrome scale was conducted unless an association was found for the respective CBCL internalizing or externalizing broadband scales. Mediation analyses were conducted using linear regression, which exhibited results similar to the main results using Poisson regression. Scores of child mental health problems and sum scores of childhood adversities were square root transformed, to approximate a normal distribution and improve linear regression model fits.

#### SNP heritability of childhood adversity

Child SNP heritability was estimated for total adversities, person-related, environment-related and adversities occurring before or after age five years. In the sample with genotype and childhood adversity, the variance explained by additive effects of autosomal SNPs was estimated using Genome-based Restricted Maximum Likelihood (GREML) as implemented in Genome-wide Complex Trait Analysis (GCTA, version 1.24.7) [35]. We used the conventional genetic relatedness matrix cut-off of 0.025 to reduce confounding due to shared environment by exclusion of close relatives (second-degree cousins and closer) as previously described [35, 36], thereby resulting in a sample of *N* = 1833 children.

## Results

### Sample characteristics

Demographic characteristics of the sample are demonstrated in Table 1. The mean age of the sample was 9.69 years (SD 0.26). The majority of children in the Generation R cohorts had not encountered any childhood adversities (*n* = 1343, 70.6%), whereas *n* = 450 (23.7%) experienced one or two adversities, and *n* = 108 (5.7%) experienced more than two adversities. The prevalence of each queried individual event is provided in Supplementary Table S1. Exposure to inappropriate sexual behaviour (0.2%) and death of a caretaker (0.3%) were the least often reported.

**Table 1:** Descriptive characteristics of the study sample

	*N*	Total population (*N* = 1901)
*Child characteristics*		
Age, mean (SD)	1901	9.69 (0.26)
Sex, proportion of girls	1901	50.3%
Childhood adversities, proportion		
No adversities	1343	70.7%
1 or 2 adversities	450	23.7%
3 or more adversities	108	5.7%
Adversities before age 5 years	185	9.7%
Adversities after age 5 years	496	26.1%
Total internalizing problems score, median (IQR)	1901	3.00 (5.00)
Total externalizing problems score, median (IQR)	1901	2.00 (5.00)
*Maternal characteristics*		
Educational level, proportion		
High	1327	73.4%
Medium	476	26.3%
Low	5	0.3%

*Note.* See Supplementary Table S1 for the prevalence of the individual adversities

### SNP-heritability of childhood adversity

The SNP heritability of total childhood adversity was 23% (SE = 0.18, *P* = 0.09). For person-related adversities, the SNP heritability was 34% (SE = 0.19, *P* = 0.03), and 6% (SE = 0.18, *P* = 0.36) for external adversities.

### Association between schizophrenia polygenic risk and childhood adversity

Child schizophrenia polygenic risk was associated with the total burden of childhood adversity (*P*_t_ < 0.5: OR = 1.08, 95% CI 1.02–1.15, *P* = 0.01; Table 2, Supplementary Table S2). Sensitivity analyses with additional adjustment for CBCL total problems scores at age 3 years yielded comparable results (*P*_t_ < 0.5: OR = 1.06, 95% CI 1.00–1.14, *P* = 0.04; Supplementary Table S3). A similar association between schizophrenia polygenic risk and childhood adversities was also present in the ALSPAC cohort (*P*_t_ < 0.5: OR = 1.02, 95% CI 1.01–1.03, *P* = 0.001; Table 2, Supplementary Table S4). No association of schizophrenia polygenic risk with adversities was found when we categorized adversities as person-related or environment-related (Supplementary Table S6). However, schizophrenia polygenic risk was associated with a higher burden of childhood adversities occurring before age 5 years (*P*_t_ < 0.5: OR = 1.20, 95% CI 1.05–1.36, *P* < 0.01; Table 2 and Supplemental Table S7). In contrast, no association of schizophrenia polygenic risk was obtained for adversities occurring after age 5 years (*P*_t_ < 0.5: OR = 1.05, 95% CI 0.98–1.13, *P* = 0.13). In the Generation R Study, no such association of the depression polygenic risk with childhood adversity was observed, and therefore no sensitivity analyses were performed (Supplementary Table S8).

**Table 2:** Association of the schizophrenia and major depression polygenic risk scores with childhood adversities in the Generation R Study (*N* = 1901)

	Childhood adversities		
	OR (95% CI)	*P*	*P*_FDR_
Generation R study (*N* = 1901)			
Total adversities	1.08 (1.02–1.15)	0.01	–
Person-related adversities	1.06 (0.97–1.16)	0.18	0.24
Environment-related adversities	1.01 (0.90–1.14)	0.87	0.87
Adversities before age 5 years	1.20 (1.05–1.36)	0.01	0.04
Adversities after age 5 years	1.05 (0.98–1.13)	0.13	0.24
Major depression risk score			
Total adversities	1.03 (0.97–1.10)	0.33	–
ALSPAC study (*N* = 3641)			
Total adversities	1.02 (1.01;1.03)	< 0.01	–

*Note*. Analyses are adjusted for age, child sex, and four principal components of genetic ancestry. Results are shown for the *P*_t_ < 0.5 inclusion threshold. Results for the other *P*-value thresholds are shown Supplementary Material

### Association between schizophrenia polygenic risk and mental health problems through childhood adversity

Given the finding of an association between schizophrenia polygenic risk and childhood adversity, we sought to assess whether the previously reported association between schizophrenia polygenic risk and childhood mental health problems, which was found in the Generation R Study [10], might be mediated by childhood adversity. In the Generation R Study, childhood adversity occurring before age ten years significantly explained part of the associations between schizophrenia polygenic risk and internalizing problems, anxious depressed problems, somatic complaints, thought problems, and attention problems (Table 3). The proportion of these mediations were 22% (95% CI -1; 65%), 23% (95% CI 0; 77%), 19% (95% CI -2; 83%), 14% (95% CI 0; 34%) and 19% (95% CI 1; 54%), respectively. However, confidence intervals were wide and in some cases overlapped with zero. Associations of schizophrenia polygenic risk with withdrawn/depressed, externalizing, and social problems were not statistically significant in the total effect models. Similar mediation estimates were observed for adversities occurring before age five years (Table 4). In the ALSPAC cohort, the previously reported association between higher schizophrenia polygenic risk and lower prosocial behaviour [7] was partly explained (5%) by exposure to childhood adversities (Table 3).

**Table 3:** The mediating effect of childhood adversity in the association between the schizophrenia polygenic risk score and childhood problem behaviour

Outcome	Total effect			Direct effect		Mediated effect		Proportion mediated	
	β (95% CI)	*P*	*P*_FDR_	β (95% CI)	*P*	β (95% CI)	*P*	Estimate (95% CI)	*P*
Generation R Study (*N* = 1901)									
Internalizing problems	0.06 (0.02;0.11)	0.01	–	0.05 (0.01;0.09)	0.02	0.01 (0.00;0.03)	0.05	0.22 (-0.01;0.65)	0.06
Anxious/depressed	0.06 (0.02;0.10)	0.01	0.02	0.05 (0.00;0.09)	0.03	0.01 (0.00;0.03)	0.05	0.23 (0.00;0.77)	0.05
Withdrawn/depressed	0.00 (− 0.04;0.04)	0.97	0.97	NA	NA	NA			
Somatic complaints	0.05 (0.01;0.10)	0.02	0.03	0.04 (0.00;0.09)	0.06	0.01 (0.00;0.02)	0.04	0.19 (− 0.02;0.83)	0.06
Externalizing problems	0.04 (0.00;0.09)	0.06	–	NA	NA	NA			
Other problems scales									
Social problem	0.04 (− 0.01;0.08)	0.11	0.13	NA	NA	NA			
Thought problems	0.08 (0.04;0.12)	< 0.01	< 0.01	0.07 (0.03;0.11)	< 0.01	0.01 (0.00;0.02)	0.05	0.14 (0.00;0.34)	0.05
Attention problems	0.07 (0.02;0.11)	< 0.01	< 0.01	0.05 (0.01;0.10)	0.01	0.01 (0.00;0.02)	0.04	0.19 (0.01;0.54)	0.04
ALSPAC cohort (*N* = 3447)									
SDQ Prosocial behaviour	− 0.05 (− 0.09;− 0.02)	< 0.01	–	− 0.05 (− 0.08;− 0.02)	< 0.01	− 0.003 (− 0.01;0.00)	0.01	0.05 (0.01;0.16)	0.01

*Note.* Analyses are adjusted for age, child sex, and four principal components of genetic ancestry. Results are shown for the *P*_t_ < 0.5 inclusion threshold

*NA*  not applicable (due to the fact that the result was not significant in the total effect analyses)

**Table 4:** The mediating effect of childhood adversity before age 5 years in the association between the schizophrenia polygenic risk score and childhood problem behaviour

Outcome	Total effect			Direct effect		Mediated effect		Proportion mediated	
	β (95% CI)	*P*	*P*_FDR_	β (95% CI)	*P*	β (95% CI)	*P*	Estimate (95% CI)	*P*
Internalizing problems	0.05 (0.01;0.10)	0.03	–	0.04 (0.00;0.08)	0.05	0.01 (0.00;0.02)	0.02	0.22 (0.04;0.87)	0.03
Anxious/depressed	0.06 (0.01;0.10)	0.01	0.02	0.05 (0.00;0.09)	0.04	0.01 (0.00;0.02)	0.01	0.19 (0.04;0.73)	0.02
Withdrawn/depressed	0.00 (− 0.04;0.05)	0.95	0.95	NA	NA	NA			
Somatic complaints	0.05 (0.01;0.10)	0.02	0.03	0.04 (0.00;0.09)	0.05	0.01 (0.00;0.02)	0.01	0.15 (0.02;0.70)	0.03
Externalizing problems	0.03 (− 0.01;0.07)	0.17	–	NA	NA	NA			
Other problems									
Social problems	0.04 (-0.01;0.08)	0.10	0.12	NA	NA	NA			
Thought problems	0.07 (0.03;0.12)	< 0.01	< 0.01	0.06 (0.02;0.11)	0.01	0.01 (0.00;0.02)	0.01	0.14 (0.03;0.40)	0.01
Attention problems	0.07 (0.02;0.11)	< 0.01	< 0.01	0.06 (0.01;0.10)	0.01	0.01 (0.00;0.02)	0.02	0.14 (0.03;0.43)	0.02

*Note*. Analyses are adjusted for age, child sex, and four principal components of genetic ancestry. Results are shown for the *P*_t_ < 0.5 inclusion threshold

*NA*  not applicable (due to the fact that the result was not significant in the total effect analyses)

## Discussion

This is the first study to explore whether the association between a child’s schizophrenia polygenic risk and mental health problems in the general population can be explained by childhood adversity. Confirming previous work, we demonstrate that increased schizophrenia polygenic risk of the child is associated with a greater exposure to childhood adversity in a population-based cohort and replicated this in an independent sample. We highlight four observations. First, we found that schizophrenia polygenic risk is associated with greater exposure to childhood adversity, an association predominantly driven by events occurring before five years of age. Second, the association of schizophrenia polygenic risk with childhood adversity partly drives the association of schizophrenia polygenic risk with childhood behaviour. Third, we obtained suggestive evidence for SNP-heritability of childhood adversity, with the largest heritability estimates for person-related adversities, consistent with a genetic association of a child’s risk of exposure to early-life adversity. Fourth, no association was observed between the major depression polygenic risk score and childhood adversity, providing some specificity to the findings using the schizophrenia polygenic risk. Together, these findings contribute to a better understanding of how gene–environment interplay might be shaping mental health problems in children.

Our findings demonstrate that common genetic variants associated with schizophrenia, as captured with a polygenic risk score, increased the odds for exposure to childhood adversity. The strongest effects were observed for adversities occurring before age five years. This could potentially be explained by that higher child polygenic risk reflects stronger effects of parental genetic factors at younger versus older child ages, although measurement factors related to retrospective recall might also play an important role [37]. In addition, given the intrinsically high co-variance of offspring polygenic scores with that of their parents, a child’s polygenic risk score effectively serves as a proxy for their parents’ genotype [22]. However, when a child is younger, the contribution of their parentally-determined environment is likely to be stronger than when children are older [38]. Therefore, we hypothesize that the relative contribution of genetic risk for environmental exposures such as childhood adversity might be age dependent. This is also corroborated by our observation of a trend towards higher genetic heritability for adversities before versus after age five years. SNP-heritability estimates of childhood traits in general population samples are commonly lower than twin-based heritability estimates [39], which might explain our suggestive finding. No distinct associations were found for person-related or environment-related events, which is likely due to insufficient power, although the odds ratio for person-related adversities was larger than the estimate for environment-related adversities.

Gene–environment correlations have received much less attention in the developmental psychopathology literature than gene–environment interactions, but it is now recognized that gene–environment correlations can substantially influence the estimation of risk between a genetic variant and a given outcome, as well as between an environmental factor and a given outcome [13, 17, 18, 40, 41]. This could occur in the context of confounding, in which the risk of a certain environmental exposure on psychopathology is partly explained by genetic effects [21]. Alternatively, the relationship between genotype and psychopathology could be mediated by environmental factors [16], such as what we observed in the current study. A child’s genotype might, for example, result in behaviours that elicit specific responses from parents, such as punishment, resulting in a greater likelihood of experiencing adversity. Similarly, higher polygenic risk for schizophrenia has recently been related to greater exposure to physical abuse in patients with first episode psychosis [42] and young people from the general population [22]. Thus, it seems reasonable to assume that a child’s genetic risk is reflective of the parents’ genetic risk for behaving in a manner that increases the frequency and/or severity of stressful adversities of the child. Such gene–environment correlations raise important issues for the design and interpretation of future etiologic studies of genetic and environmental risks, in particular those involving childhood cohorts.

We quantified a 34% SNP-heritability for person-related adversities, which, among others, comprised maltreatment, school problems and family conflict. In contrast, SNP-heritability for environment-related adversities was much smaller and compatible with the null hypothesis. An estimation of 34% SNP-heritability is relatively high compared to other such heritability estimates of childhood traits in the general population [39, 43], which may simply reflect the wide confidence intervals due to limited sample size. However, this finding is consistent with our interpretation of a gene–environment correlation through childhood adversities. We posit that this reflects a child’s genetic vulnerability to schizophrenia, which makes the child more likely to be the recipient of adversities imposed upon him or her through the behaviour of parents or others (i.e. passive or evocative gene–environment correlation) [20, 40]. Future studies incorporating child, maternal and paternal genotyping, as well as large adoption studies would have the potential to distinguish between passive versus evocative gene–environment correlation [22, 44–46]. Alternatively, children with an increased genetic risk for schizophrenia might experience greater adversity as a consequence of their own behaviours (i.e. active gene–environment correlation). In short, although a causal mediation of schizophrenia polygenic risk with behaviour through adverse life events cannot be fully excluded, we argue that the associations in the current study can be interpreted best as gene–environment correlations. Therefore, although several mechanisms could explain the association between schizophrenia polygenic risk and childhood adversities (Fig. 1), other methods need to be employed to examine its causal nature and relationship with mediators, such as multivariable Mendelian Randomisation with mediation analyses [47].

Several previous studies have employed data on familial history of schizophrenia to study the interplay between genetic and environmental risks. However, measures of familial risk are crude estimates of genetic liability as outcomes may be poorly recorded or remembered and the absence of a known family history might not adequately reflect heritable factors [48]. In the current study, we used polygenic risk scores as an additive genetic risk metric for schizophrenia, which is a more generalizable, widely applicable and continuous measure of genetic liability than family history. This in turn increases the power to examine subtle effects in general population samples. However, it should be noted that SNP-heritability and polygenic risk scores assume additivity of the individual SNPs captured by the risk score. Accordingly, it might be more biologically informative to examine differential gene–environment susceptibility using biologically informed polygenic risk scoring employing weighting based on defined cell-types implicated in the aetiology of a disease or trait [49].

Sub-clinical psychiatric manifestations of emotional and behavioural problems are common in non-selected paediatric samples from the general population. These symptoms are more common in children with higher schizophrenia polygenic risk scores and predictive of future clinical disorders [6–8, 10], consistent with the higher prevalence of psychiatric illness among offspring of parents with severe mental illness [48]. In line with our earlier work [10], we observed associations of schizophrenia polygenic risk with emotional, attention and thought problems, while no associations were obtained with behavioural problems. This suggests a particular involvement of emotional problems as phenotypic expressions of elevated genetic vulnerability for schizophrenia in pre-adolescent children [6, 7, 10, 50].

Although the current study was population-based, our findings could have important potential clinical implications. Given that the pathway from increased genetic vulnerability for schizophrenia to phenotypic manifestations of mental health problems was partially explained by childhood adversities, future studies are warranted to consider whether counselling of children at high genetic risk and their caretakers might offer an opportunity for attenuating the risk of subsequent conversion to a clinical mental health disorder. Stable relationships between intimate partners and between mothers and their children have been associated with breaking the intergenerational transmission of abuse in families [51], which, given these and our observations, should be further explored in children growing up in families at high genetic risk for severe mental illness. Parental psychopathology affects the ability of parents to provide stable and nurturing environments for children [52]. Therefore, early (i.e. preconceptional and perinatal) support and education about lifestyle and parenting skills are paramount [51].

Our study was characterized by several strengths, including its prospective population-based design and replication in an independent prospective birth cohort. We also included a comprehensive interview for childhood adversities to assess the timing and impact of each reported event. However, several limitations need to be discussed. First, we did not have bi-parental genotype data available to disentangle whether the genetic factors predicting childhood adversities were disproportionally located on transmitted and/or non-transmitted alleles [44], however, recent work in the ALSPAC cohort has clearly demonstrated the importance of parental genotype in the context of genetic risk for schizophrenia and exposure to childhood trauma [22]. Second, assessment of childhood adversities and child mental health problems relied on maternal report in both cohorts, which could have introduced shared reporter bias. Third, assessments of exposure to adversities in the Generation R cohort could have been biased by retrospective reporting as mothers might disproportionately remember adversities of higher severity with increasing passage of time. However, in the ALSPAC study we found very comparable results with a prospective assessment of stressful life events. Fourth, although we obtained evidence for mediation in this study, this does not necessarily infer causality [53]. Rather, our mediation analysis provides support for gene–environment correlation in the context of genetic vulnerability for schizophrenia. And finally, in line with the majority of studies employing polygenic risk score methods [54], the associations of schizophrenia polygenic risk with childhood adversities and mental health problems were of small effect and not found across all polygenic risk score thresholds, tempering definitive conclusions about our findings. However, considering that polygenic risk scores explain very little of the variation in the original phenotype (approximately 7%), the small effect sizes could potentially result from these ceiling effects by design. Therefore, any replicated association with another phenotype and in an independent sample is noteworthy.

In summary, we observed that elevated genetic risk for schizophrenia, as quantified by polygenic risk, is associated with higher exposure to childhood adversity. Childhood adversity partly explained the relationship between schizophrenia genetic liability and mental health problems in childhood, providing evidence of gene–environment correlation. Hence, these findings suggest the need to consider the benefits and risks of preventative measures aimed at reducing exposure to adversity in early childhood.

## Supplementary Information

Below is the link to the electronic supplementary material.

Supplementary file1 (DOC 179 KB)

## Data Availability

Requests for data can be made through Prof. Dr. Vincent Jaddoe, lead investigator of the Generation R Study.
