# Supplementary material for: Schizophrenia polygenic risk is associated with child mental health problems through early childhood adversity: evidence for a gene–environment correlation
Source: Eur Child Adolesc Psychiatry. 2021 Feb 26;31(3):529–39. doi: 10.1007/s00787-021-01727-4 (PMC8940779; doi:10.1007/s00787-021-01727-4)
Supplement: Supplementary file 1 — Supplementary file1 (DOC 179 KB) [file 787_2021_1727_MOESM1_ESM.doc]

**Supplementary material**

**Replication sample**

A total of 9,912 subjects were genotyped using the Illumina HumanHap550 quad genome-wide SNP genotyping platform by 23andMe subcontracting the Wellcome Trust Sanger Institute, Cambridge, UK and the Laboratory Corporation of America, Burlington, NC, USA. Individuals were excluded from further analysis on the basis of having incorrect gender assignments; minimal or excessive heterozygosity (<0·320 and >0·345 for the Sanger data and <0·310 and >0·330 for the LabCorp data); disproportionate levels of individual missingness (>3%); evidence of cryptic relatedness (>10% IBD) and being of non-European ancestry as detected by a multidimensional scaling analysis seeded with HapMap 2 individuals in which EIGENSTRAT analysis revealed no additional obvious population stratification and genome-wide analyses with other phenotypes indicated a low lambda. SNPs with a minor allele frequency of <1% and call rate of <95% were removed. Furthermore, only SNPs which passed an exact test of Hardy–Weinberg equilibrium (P>5×10^-7^) were considered for analysis. After quality control, 8365 unrelated individuals who were genotyped at 500527 SNPs, were available for analysis. EIGENSTRAT principal components analysis was used to generate the top 100 principal components after the removal of known regions of long linkage disequilibrium in the data.^8,9^ Known autosomal variants were imputed with MACH 1.0.16 Markov Chain Haplotyping software,^10,11^ using CEPH individuals from phase 2 of the HapMap project (hg18) as a reference set (release 22). In total, 3641children had complete data on cumulative childhood life events until age 9 years. Childhood adversities were assessed at ages 8 weeks, 8 months, 21 months, 3 years, 4 years, 5 years, 6 years, and 9 years of age using the mother-reported Life Events Scale,^12^ of which individual total scores were summed to generate a cumulative childhood adversity score. Of these, 3447 children had complete data on the Strengths and Difficulties Questionnaire (SDQ^13^), which is a brief questionnaire to assess common mental health difficulties in children. The scale was parent-reported when the child was age 10 years, and comprised the subscales prosocial behaviour, emotional problems, conduct problems, and hyperactivity problems. Given that a previously study reported a negative association between schizophrenia polygenic risk and the SDQ prosocial behaviour scale (but not other SDQ scales),^14^ the present analyses will be limited to SDQ prosocial behaviours. A similar negative association was observed in the current study (Supplementary Table S4).

**Supplementary Table S1: Overview of the childhood adversities ascertained through mother interview.**

| **#** | **Item** | **Prevalence** | **Category** |
| --- | --- | --- | --- |
| 1 | Did your child get seriously sick or did he/she have an accident? | 2·9 | Person |
| 2 | Did a family member get seriously sick or did someone have a serious accident? | 3·3 | Environment |
| 3 | Did someone else, who is important to the child, get seriously sick or did someone have a serious accident? | 4·0 | Environment |
| 4 | Has the father/mother or other caretaker of your child died? | 0·3 | Environment |
| 5 | Has someone else, who your child cared a lot about, passed away? | 4·2 | Environment |
| 6 | Has a pet, who you child cared a lot about, die? | 2·1 | Environment |
| 7 | Does or did your child have to deal with a high workload at school? | 9·5 | Person |
| 8 | Has your child ever repeated a grade? | 1·0 | Person |
| 9 | Are/were there any neighbourhood problems? E.g. vandalism or insecurity. | 1·8 | Environment |
| 10 | Has your family financial difficulties or had your family ever have them? | 1·5 | Environment |
| 11 | Does your child have ongoing conflicts with a family member (or did your child ever have them)? | 2·0 | Person |
| 12 | Does your child have ongoing conflicts with someone else (or did your child ever have them)? | 2·7 | Person |
| 13 | Do other family member have ongoing conflicts with each other (or did they ever have them)? | 3·2 | Environment |
| 14 | Are you and your partner divorced or separated? | 4·8 | Environment |
| 15 | Did one of the parents become involuntarily unemployed? | 0·9 | Environment |
| 16 | Did your child lose a good friend due to an argument? | 0·8 | Person |
| 17 | Did your child every lose something which was important to him/her? E.g. through fire, loss, or theft. | 1·0 | Person |
| 18 | Has someone ever used physical violence against your child? For example, beating him/her up. | 0·9 | Person |
| 19 | Has someone almost used physical violence against your child? So that not actually happened, but your child was frightened. | 1·4 | Person |
| 20 | Has someone made sexual comments or movements towards your child? | 0·5 | Person |
| 21 | Has your child experienced inappropriate sexual behavior? | 0·2 | Person |
| 22 | Has someone spread mean rumours about your child? | 1·9 | Person |
| 23 | Has your child moved to a different place of residence? | 1·2 | Environment |
| 24 | Has your child changed schools? | 1·5 | Environment |

**Supplemental Table S2**: Association between the schizophrenia polygenic risk score and childhood adversities in the Generation R Study (*N* = 1901).

|  | **Childhood adversities** | |
| --- | --- | --- |
| **Polygenic risk score threshold** | **OR (95% CI)** | ***P*** |
| P < 0·0005 | 1·00 (0·94;1·06) | 1·00 |
| P < 0·001 | 1·00 (0·94;1·07) | 0·90 |
| P < 0·005 | 1·00 (0·94;1·06) | 0·89 |
| P < 0·01 | 1·02 (0·96;1·08) | 0·59 |
| P < 0·05 | 1·04 (0·97;1·11) | 0·25 |
| P < 0·1 | 1·07 (1·00;1·14) | 0·04 |
| P < 0·5 | 1·08 (1·02;1·15) | 0·01 |
| P < 1·0 | 1·09 (1·02;1·16) | 0·01 |

Note: Analyses are adjusted for age, child sex, and four principal components of genetic ancestry.

**Supplemental Table S3**: Association between the schizophrenia polygenic risk score and childhood adversities in the Generation R Study (*N* = 1901), with additional adjustment for CBCL total problems scores at age 3 years.

|  | **Childhood adversities** | |
| --- | --- | --- |
| **Polygenic risk score threshold** | **OR (95% CI)** | ***P*** |
| P < 0·0005 | 1·00 (0·94;1·06) | 1·00 |
| P < 0·001 | 1·00 (0·94;1·07) | 0·92 |
| P < 0·005 | 0·99 (0·93;1·05) | 0·77 |
| P < 0·01 | 1·01 (0·95;1·07) | 0·78 |
| P < 0·05 | 1·02 (0·96;1·09) | 0·47 |
| P < 0·1 | 1·05 (0·99;1·12) | 0·10 |
| P < 0·5 | 1·07 (1·00;1·14) | 0·04 |
| P < 1·0 | 1·07 (1·01;1·14) | 0·03 |

Note: Analyses are adjusted for age, child sex, four principal components of genetic ancestry, and CBCL total problems scores at age 3 years.

**Supplemental Table S4a (Poisson regression): Association between the schizophrenia polygenic risk score and life events child scale in the ALSPAC cohort (*N* = 3671).**

|  | **Childhood adversities** | |
| --- | --- | --- |
| **Polygenic risk score threshold** | **OR (95% CI)** | ***P*** |
| P < 0·01 | 1·02 (1·01;1·03) | <0·01 |
| P < 0·05 | 1·02 (1·01;1·03) | <0·01 |
| P < 0·1 | 1·02 (1·01;1·03) | <0·01 |
| P < 0·2 | 1·02 (1·01;1·03) | <0·01 |
| P < 0·3 | 1·02 (1·01;1·03) | <0·01 |
| P < 0·4 | 1·02 (1·01;1·03) | <0·01 |
| P < 0·5 | 1·02 (1·01;1·03) | <0·01 |
| P < 1·0 | 1·02 (1·01;1·03) | <0·01 |

Note: Analyses are adjusted for child sex, and four principal components of genetic ancestry.

**Supplemental Table S4b (linear regression)**: Association between the schizophrenia polygenic risk score and life events child scale in the ALSPAC cohort (*N* = 3671).

|  | **Childhood adversities** | |
| --- | --- | --- |
| **Polygenic risk score threshold** | **β (95% CI)** | ***P*** |
| P < 0·01 | 0·04 (0·00;0·07) | 0·03 |
| P < 0·05 | 0·04 (0·01;0·07) | 0·02 |
| P < 0·1 | 0·04 (0·00;0·07) | 0·03 |
| P < 0·2 | 0·04 (0·00;0·07) | 0·03 |
| P < 0·3 | 0·04 (0·01;0·07) | 0·02 |
| P < 0·4 | 0·04 (0·01;0·07) | 0·02 |
| P < 0·5 | 0·04 (0·01;0·07) | 0·01 |
| P < 1·0 | 0·04 (0·01;0·07) | 0·02 |

Note: Analyses are adjusted for child sex, and four principal components of genetic ancestry.

**Supplemental Table S5**: Association between the schizophrenia polygenic risk score and SDQ prosocial behaviour in the ALSPAC cohort (*N* = 3447).

|  | **Prosocial behaviour** | |
| --- | --- | --- |
| **Polygenic risk score threshold** | **β (95% CI)** | ***P*** |
| P < 0·01 | -0·05 (-0·08;-0·01) | <0·01 |
| P < 0·05 | -0·06 (-0·10;-0·03) | <0·01 |
| P < 0·1 | -0·05 (-0·09;-0·02) | <0·01 |
| P < 0·2 | -0·06 (-0·09;-0·03) | <0·01 |
| P < 0·3 | -0·05 (-0·09;-0·02) | <0·01 |
| P < 0·4 | -0·05 (-0·09;-0·02) | <0·01 |
| P < 0·5 | -0·05 (-0·09;-0·02) | <0·01 |
| P < 1·0 | -0·05 (-0·08;-0·02) | <0·01 |

Note: Analyses are adjusted for age, child sex, and four principal components of genetic ancestry.

**Supplemental Table S6**: Association between schizophrenia polygenic risk scores and childhood adversity, divided in events before and after age 5 years in the Generation R Study (*N* = 1901).

|  | Childhood adversities | | | |
| --- | --- | --- | --- | --- |
|  | Before age 5 years | | After age 5 years | |
| Polygenic risk score threshold | OR (95% CI) | *P* | OR (95% CI) | *P* |
| P < 0·0005 | 1·12 (0·98;1·27) | 0·10 | 0·98 (0·92;1·05) | 0·56 |
| P < 0·001 | 1·08 (0·95;1·23) | 0·22 | 0·99 (0·92;1·06) | 0·70 |
| P < 0·005 | 1·15 (1·01;1·31) | 0·03 | 0·96 (0·90;1·03) | 0·26 |
| P < 0·01 | 1·18 (1·04;1·35) | 0·01 | 0·98 (0·91;1·05) | 0·54 |
| P < 0·05 | 1·20 (1·06;1·36) | 0·01 | 1·00 (0·94;1·07) | 0·96 |
| P < 0·1 | 1·20 (1·06;1·37) | <0·01 | 1·03 (0·97;1·11) | 0·34 |
| P < 0·5 | 1·20 (1·05;1·36) | 0·01 | 1·05 (0·98;1·13) | 0·13 |
| P < 1·0 | 1·20 (1·06;1·36) | 0·01 | 1·06 (0·99;1·14) | 0·10 |

Note: Analyses are adjusted for age, child sex, and four principal components of genetic ancestry.

**Supplemental Table S8**: Association between the major depressive disorder polygenic risk score and childhood adversities in the Generation R Study (*N* = 1901).

|  | Childhood adversities | |
| --- | --- | --- |
| Polygenic risk score threshold | OR (95% CI) | *P* |
| P < 0.0005 | 0.98 (0.92–1.05) | 0.58 |
| P < 0.001 | 0.96 (0.90–1.02) | 0.23 |
| P < 0.005 | 1.02 (0.96–1.09) | 0.50 |
| P < 0.01 | 1.01 (0.95–1.08) | 0.73 |
| P < 0.05 | 1.01 (0.95–1.07) | 0.82 |
| P < 0.1 | 1.03 (0.97–1.10) | 0.37 |
| P < 0.5 | 1.03 (0.97–1.10) | 0.33 |
| P < 1.0 | 1.04 (0.98–1.11) | 0.24 |

Note: Analyses are adjusted for age, child sex, and four principal components of genetic ancestry.

**Supplemental Figure S1**: Inclusion flowchart of the study population


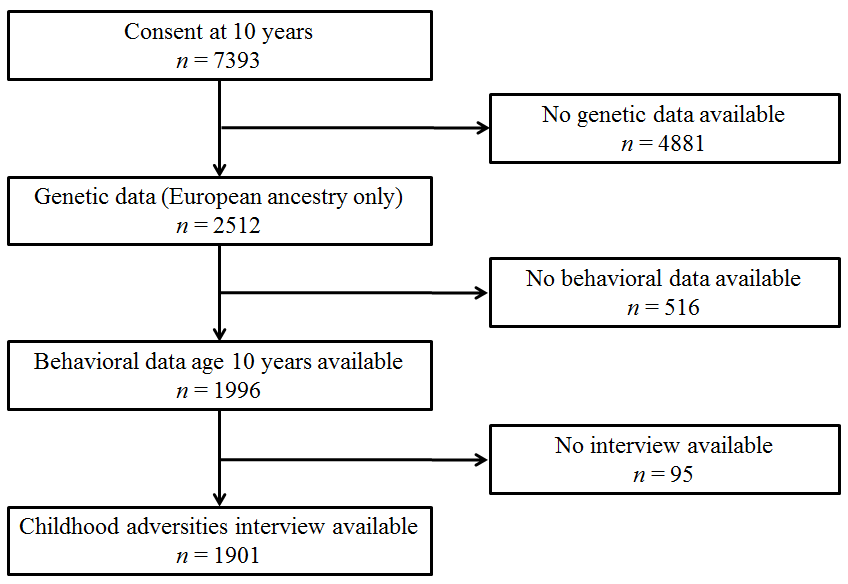


**References**

1. Medina-Gomez C, Felix JF, Estrada K, et al. Challenges in conducting genome-wide association studies in highly admixed multi-ethnic populations: the Generation R Study. *Eur J Epidemiol* 2015; **30**(4): 317-30.

2. Delaneau O, Marchini J, Zagury JF. A linear complexity phasing method for thousands of genomes. *Nat Methods* 2011; **9**(2): 179-81.

3. Howie B, Fuchsberger C, Stephens M, Marchini J, Abecasis GR. Fast and accurate genotype imputation in genome-wide association studies through pre-phasing. *Nat Genet* 2012; **44**(8): 955-9.

4. Schizophrenia Working Group of the Psychiatric Genomics Consortium. Biological insights from 108 schizophrenia-associated genetic loci. *Nature* 2014; **511**(7510): 421-7.

5. Wray NR, Ripke S, Mattheisen M, et al. Genome-wide association analyses identify 44 risk variants and refine the genetic architecture of major depression. *Nat Genet* 2018.

6. Jansen PR, Polderman TJC, Bolhuis K, et al. Polygenic scores for schizophrenia and educational attainment are associated with behavioural problems in early childhood in the general population. *J Child Psychol Psychiatry* 2018; **59**(1): 39-47.

7. Euesden J, Lewis CM, O'Reilly PF. PRSice: Polygenic Risk Score software. *Bioinformatics* 2015; **31**(9): 1466-8.

8. Price AL, Patterson NJ, Plenge RM, Weinblatt ME, Shadick NA, Reich D. Principal components analysis corrects for stratification in genome-wide association studies. *Nat Genet* 2006; **38**(8): 904-9.

9. Price AL, Weale ME, Patterson N, et al. Long-range LD can confound genome scans in admixed populations. *Am J Hum Genet* 2008; **83**(1): 132-5; author reply 5-9.

10. Li Y, Willer C, Sanna S, Abecasis G. Genotype imputation. *Annu Rev Genomics Hum Genet* 2009; **10**: 387-406.

11. Li Y, Willer CJ, Ding J, Scheet P, Abecasis GR. MaCH: using sequence and genotype data to estimate haplotypes and unobserved genotypes. *Genet Epidemiol* 2010; **34**(8): 816-34.

12. Cecil CA, Lysenko LJ, Jaffee SR, et al. Environmental risk, Oxytocin Receptor Gene (OXTR) methylation and youth callous-unemotional traits: a 13-year longitudinal study. *Mol Psychiatry* 2014; **19**(10): 1071-7.

13. Goodman R. The Strengths and Difficulties Questionnaire: a research note. *J Child Psychol Psychiatry* 1997; **38**(5): 581-6.

14. Riglin L, Collishaw S, Richards A, et al. Schizophrenia risk alleles and neurodevelopmental outcomes in childhood: a population-based cohort study. *Lancet Psychiatry* 2017; **4**(1): 57-62.
